# Microsatellite marker development for the tetraploid *Veronica aragonensis* (Plantaginaceae) using next‐generation sequencing and high‐resolution melting analyses

**DOI:** 10.1002/aps3.1154

**Published:** 2018-06-05

**Authors:** Nélida Padilla‐García, Teresa Malvar‐Ferreras, Josie Lambourdière, M. Montserrat Martínez‐Ortega, Nathalie Machon

**Affiliations:** ^1^ Departamento de Botánica Universidad de Salamanca E‐37007 Salamanca Spain; ^2^ Biobanco de ADN Vegetal Universidad de Salamanca Edificio Multiusos I+D+i, Calle Espejo s/n 37007 Salamanca Spain; ^3^ Centre d'Ecologie et des Sciences de la Conservation (CESCO, UMR7204) Sorbonne Université, MNHN, CNRS, UPMC CP51 Paris France; ^4^ Service de Systématique Moléculaire (UMS 2700) Sorbonne Université, CNRS‐MNHN 43 rue Cuvier 75005 Paris France

**Keywords:** high‐resolution melting (HRM) analyses, microsatellites, Plantaginaceae, polyploidy, *Veronica aragonensis*

## Abstract

**Premise of the Study:**

The tetraploid *Veronica aragonensis* (Plantaginaceae) is a narrow endemic to the Iberian Peninsula. Specific microsatellite markers were developed to investigate genetic structure and diversity.

**Methods and Results:**

A total of 15 polymorphic markers were characterized on three populations of *V. aragonensis*, using a microsatellite‐enriched library on an Ion Torrent sequencer and high‐resolution melting (HRM) analyses to rapidly discard nonreliable, multicopy, and/or monomorphic loci. Allele number per locus ranged from one to five, and levels of observed heterozygosity per population varied from 0.142 ± 0.301 to 0.281 ± 0.369. Most primers also amplified in the closely related species *V. rosea* and in three subspecies of *V. tenuifolia*.

**Conclusions:**

The species‐specific microsatellite markers developed here represent an essential tool to provide genetic information on the population level for *V. aragonensis*. The low levels of variation detected highlight the importance of continued efforts to improve conservation of the species.

*Veronica aragonensis* Stroh (Plantaginaceae) is a perennial herb included in the diploid–polyploid complex *Veronica* subsect. *Pentasepalae* Benth., one of the four subsections recognized within *Veronica* subgen. *Pentasepalae* M. M. Mart. Ort., Albach & M. A. Fisch. (Rojas‐Andrés et al., 2015). This endemic plant is restricted to three disjunct mountain areas in the Iberian Peninsula (Martínez‐Ortega et al., 2009). It is one of the few highly specialized plants growing in Iberian limestone mountain screes (between 1000 and 2300 m). Given that it is a rare species, it is included in regional catalogs and Red Lists from Spain (Cabezudo et al., 2005; Alcántara de la Fuente et al., 2007).

A set of microsatellite markers was previously developed for other species from *Veronica* subsect. *Pentasepalae* (i.e., *V. austriaca* L. subsp. *jacquinii* (Baumg.) Watzl and *V. orbiculata* A. Kern.; López‐González et al., 2015). However, preliminary cross‐transferability tests performed for most of these loci resulted either in monomorphic patterns or unsuccessful amplifications in *V. aragonensis* (results not shown). Successful cross‐species transfer of nuclear microsatellite markers is usually limited—particularly in terms of polymorphism—by large evolutionary distances (Ellegren et al., 1995; Barbará et al., 2007). Previous studies suggested that *V. aragonensis* is relatively isolated from the remaining species of the subsection (Martínez‐Ortega et al., 2004; Rojas‐Andrés et al., 2015; Padilla‐García et al., 2018). This may be precluding cross‐transferability success. In this situation, new microsatellite markers must be developed to address the study of gene flow patterns and genetic structure in the narrow endemic *V. aragonensis*.

## METHODS AND RESULTS

Genomic DNA from one individual of *V. aragonensis* (Appendix 1) was extracted following the cetyltrimethylammonium bromide (CTAB) protocol (Doyle and Doyle, 1987). A DNA library was generated on an Ion Torrent Personal Genome Machine Sequencer (Life Technologies, Saint Aubin, France) using the kit NEBNext Fast DNA Fragmentation & Library Prep Set for Ion Torrent (New England Biolabs, Ipswich, Massachusetts, USA). Then, an emulsion PCR was performed to enrich the library, and sequencing was performed using 800 flows (generating ca. 100–400 bp read lengths) on an Ion 316 v2 sequencing chip (Life Technologies). Sequences were submitted to the National Center for Biotechnology Information's (NCBI) Sequence Read Archive (SRA; accession no. SRP129594). BioProject information and BioSample records are available under accession numbers PRJNA429875 and SAMN08362105, respectively. From a total of 737,951 sequences, 11,604 microsatellites were detected, and 4572 of them were in singleton sequences. Microsatellite selection and primer design were performed using QDD version 3.1 (Meglécz et al., 2014) for detecting unique microsatellite sequences, with a minimum of five repeats, a PCR product size of 90–450 bp, an optimal temperature of 60°C, and 50% of GC. Primers were designed for 1727 microsatellites, of which 50 were tested for polymorphism.

High‐resolution melting (HRM) analyses were used as a previous screening to rapidly identify PCR failure, monomorphism, or multicopy status of microsatellite loci (Arthofer et al., 2011). Amplification and HRM analyses were performed on a CFX96 Real‐Time PCR Detection System (Bio‐Rad Laboratories, Hercules, California, USA) using SsoFast EvaGreen 2× SuperMix (Bio‐Rad Laboratories) with 0.4 μM simple sequence repeat (SSR)–specific primers and 2 μL of template DNA (ca. 32 ng/μL) in a 10 μL total reaction volume. Cycling conditions were 2 min initial hot start at 98°C, followed by 40 cycles of 98°C for 5 s, 60°C for 10 s, and 72°C for 20 s. Cycling was followed by 20 s holds at 95°C to ensure a homogeneous denaturation of amplicons. HRM analysis consisted of an initial 5 s hold at 65°C and ramping from 65°C to 95°C in 0.2°C steps. Each step was held for 5 s before the fluorescence was acquired. Melting‐temperature ranges and differences in curve shape among samples were analyzed as a measure of SSR size variation. Of 50 loci tested by HRM analyses, eight did not amplify in quantitative PCR and 16 were excluded as monomorphic due to the low melting temperature range observed (≤0.20 K). Although polymorphism was difficult to confirm by this methodology, it allowed us to screen for robust amplification and single‐copy status of the tested loci (Table 1).

**Table 1:** Results from high‐resolution melting analyses

Locus	Repeat motif	Product size (bp)	No. of dF/dT peaks	*T*_a_ range (K)	Variability
01	(AAT)_12_	90	—	—	No amplification
02	(AT)_12_	90	2	1.20	Potentially polymorphic
03	(AC)_11_	91	1	0.40	Potentially polymorphic
04	(AGAT)_9_	95	1	0.40*	Potentially polymorphic
05	(AAT)_13_	97	—	—	No amplification
06	(AGAT)_6_	103	—	—	No amplification
07	(AAGAC)_7_	103	1	0.40	Potentially polymorphic
08	(AT)_10_	105	—	—	No amplification
09	(AG)_13_	105	—	—	No amplification
10	(AAT)_9_	111	1–2	1.20	Potentially polymorphic
11	(AT)_10_	113	1	0.40*	Potentially polymorphic
12	(AAAC)_8_	114	1	0.60	Potentially polymorphic
13	(AC)_9_	115	1	0.40*	Potentially polymorphic
14	(AAT)_9_	126	1–2	4.00	Potentially polymorphic
15	(AC)_10_	127	1	0.20*	Potentially polymorphic
16	(AT)_15_	129	—	—	No amplification
17	(ATATC)_6_	137	1	0.80	Potentially polymorphic
18	(ACAT)_9_	138	1	0.40	Potentially polymorphic
19	(AG)_9_	138	1	0.20	Monomorphic
20	(AAT)_9_	141	1	0.40	Potentially polymorphic
21	(AAAT)_6_	150	2	2.00	Potentially polymorphic
22	(AATT)_7_	156	1	0.00	Monomorphic
23	(AAG)_10_	162	1	0.40*	Potentially polymorphic
24	(AT)_11_	162	—	—	No amplification
25	(AG)_10_	166	1	0.20*	Potentially polymorphic
26	(AAAAT)_5_	167	1–2	0.60	Potentially polymorphic
27	(AAT)_14_	180	1	0.20*	Potentially polymorphic
28	(AAAG)_6_	185	1	0.20	Monomorphic
29	(AT)_10_	190	1	0.40*	Potentially polymorphic
30	(AAAC)_6_	191	1	0.20	Monomorphic
31	(AC)_8_	191	1	0.40	Potentially polymorphic
32	(AT)_13_	195	1–2	2.80	Potentially polymorphic
33	(AAT)_18_	195	—	—	No amplification
34	(AAGG)_6_	196	1	0.40	Potentially polymorphic
35	(AC)_8_	198	1	0.40	Potentially polymorphic
36	(AT)_7_	207	2	0.20*	Potentially polymorphic
37	(AAT)_10_	207	1	0.20	Monomorphic
38	(AC)_10_	219	1	0.20	Monomorphic
39	(AATC)_10_	225	1	0.20	Monomorphic
40	(AAC)_9_	240	1	0.20	Monomorphic
41	(AC)_16_	240	1	0.60	Potentially polymorphic
42	(AT)_8_	253	1	0.20	Monomorphic
43	(AT)_11_	260	2	0.40*	Potentially polymorphic
44	(ACT)_9_	265	2	0.00	Monomorphic
45	(ACTC)_6_	270	1	0.20	Monomorphic
46	(AAAG)_6_	290	1	0.20	Monomorphic
47	(AT)_8_	297	1	0.20	Monomorphic
48	(AAAAC)_5_	340	1	0.20	Monomorphic
49	(AG)_10_	340	1	0.00	Monomorphic
50	(AT)_9_	369	1	0.00	Monomorphic

Note

— = no data due to failed PCR amplification; dF/dT peaks = peaks observed in the melt curve when plotting the derivative of fluorescence over temperature; K = melting temperature range; *T*_a_ = annealing temperature.

*Differences observed in curve shape among samples.

The remaining loci (26) were genotyped on 11 individuals from a single population of *V. aragonensis* and 10 individuals from 10 different populations (Appendix 1) to evaluate the intrapopulation and interpopulation polymorphism of the markers, respectively. PCR reactions contained 1.25 μL of *Taq* Pol Buffer (10×), 0.8 mM of dNTPs mix (Life Technologies, Carlsbad, California, USA), 1.5 mM of MgCl_2_, 0.08 μM of each forward primer modified with an M13 tail, 0.2 μM of reverse primer, 0.2 μM of fluorescent‐labeled M13 universal primer, 0.5 units *Taq* DNA Polymerase (Biotools B&M Labs S.A., Madrid, Spain), 40–50 ng of DNA template, and H_2_O up to a final volume of 12.5 μL. Gradient PCRs were performed to test all primers as follows: 2 min at 94°C; 30 cycles of 1 min at 94°C, 1 min at 55.7–62.5°C, and 50 s at 72°C; followed by 10 cycles of 1 min at 94°C, 1 min at 53°C, and 50 s at 72°C; with a final extension of 15 min at 72°C. PCR products were visualized on a 2.5% agarose gel and separated on a multi‐capillary sequencer ABI PRISM 3730 (Applied Biosystems, Waltham, Massachusetts, USA) using GeneScan 500 LIZ Size Standard (Applied Biosystems). Electropherograms were visualized and scored with GeneMarker version 1.8 software (SoftGenetics, State College, Pennsylvania, USA). Fifteen primer combinations (Table 2) displaying clear peak patterns and polymorphism were combined in multiplex reactions according to annealing temperature and amplicon sizes. Sequences from these loci were deposited in GenBank (Table 2).

**Table 2:** Description of 15 microsatellite loci developed in *Veronica aragonensis*

Locus	Primer sequences (5′–3′)	Fluorescent dye	Repeat motif	Allele size range (bp)	*T*_a_ (°C)	GenBank accession no.
04	F: TCACTGTAAACTTACCTCCCATTC	5‐FAM	(AGAT)_9_	94–126	61.2	MF946655
	R: AACACAAGAGTAGGTCGCCTG					
10	F: AGCATGACTCGGTTCATCAC	5‐FAM	(AAT)_9_	115–160	55.7	MF946656
	R: CGATATGCGTGGTAACTTGG					
11	F: CAACTGATAGAAAGAATCTGCAAC	PET	(AT)_10_	124–134	61.2	MF946657
	R: CAGGAAATCAGCCTGTGCTC					
12	F: TCAATGTCCACCTTCTGCTG	NED	(AAAC)_8_	105–125	61.2	MF946658
	R: CATTCATTCTCGTACGTTGGG					
13	F: TCCATCTTGGAATGTCCATC	VIC	(AC)_9_	127–137	61.2	MF946659
	R: CATGAACAAACATTGATTAGTAAACC					
15	F: TGAGTGGATAGAGTTGGAGGC	PET	(AC)_10_	145–157	61.2	MF946660
	R: AAGACATAATCAAGCACTAATCCTC					
21	F: TCAAGCTGTTGCCCAACTC	NED	(AAAT)_6_	169–193	61.2	MF946661
	R: CATTTCAGCTTTCATTTCATTACAG					
23	F: TTCTTCCTTCTTCGACACGG	VIC	(AAG)_10_	164–206	57.2	MF946662
	R: TTTGTCAACATATTTCAAGATCCG					
25	F: TGATTATTTACTTTAAGATTGACACCG	NED	(AG)_10_	180–206	57.2	MF946663
	R: TATGCTCTGATTCTGGACGG					
26	F: CCGTTACACTCGAAGTATCCC	VIC	(AAAAT)_5_	172–187	61.2	MF946664
	R: CGTTTAAATTGCGAGTTTGTTG					
27	F: TGCTGATTGCTGAATATTGGAC	5‐FAM	(AAT)_14_	167–225	61.2	MF946665
	R: AATCTGGGTCGTGATTCTGG					
29	F: CAGATGACTTTGACGGAGAATC	PET	(AT)_10_	205–225	61.2	MF946666
	R: TTCACTCGTATTCCTATTTCCGC					
36	F: ACAACTAACTTTGAGAAATTACCATTC	PET	(AT)_7_	226–240	61.2	MF946667
	R: ATGAGTGGCGTTAGGGTTTG					
53	F: GCTAAATAACAAACAACAAGAAAGATG	NED	(AT)_10_	104–122	55.7	MF946668
	R: TTGATGTCAGTCATAATCCACC					
56	F: AAGAGGGTTAATGGATGGTTG	VIC	(AAAG)_6_	128–148	61.2	MF946669
	R: CCAACCCTTATTCATCTAAAGTATATC					

Note

*T*_a_ = annealing temperature.

To characterize the microsatellite loci, a total of 92 individuals from three populations representing the main distribution areas of this endemic species were used (34, 23, and 35 individuals from the Nerín, Arguís, and La Sagra populations, respectively; see Appendix 1). Three loci (27, 29, and 53) did not amplify across all 92 samples, and loci 11 and 12 resulted in imperfect microsatellites. These markers were finally discarded due to difficult scoring. For the remaining 10 loci, sample size, number of alleles, observed heterozygosity, and expected heterozygosity (with and without correction of allele dosages for polyploids) were evaluated with GENODIVE (Meirmans and Van Tienderen, 2004). The number of alleles per locus ranged from one to five. Levels of observed heterozygosity (mean ± SD) were 0.246 ± 0.273, 0.281 ± 0.369, and 0.142 ± 0.301 for the Nerín, Arguís, and La Sagra populations, respectively (Table 3).

**Table 3:** Genetic characterization of 10 polymorphic microsatellites in three populations of *Veronica aragonensis*.^a^

Locus	Nerín (*N* = 34)				Arguís (*N* = 23)				La Sagra (*N* = 35)			
	*A*	*H*_o_	*H*_e_	*H*_e‐d_	*A*	*H*_o_	*H*_e_	*H*_e‐d_	*A*	*H*_o_	*H*_e_	*H*_e‐d_
04	2	0.273	0.383	0.379	3	0.000	0.372	0.372	2	0.000	0.115	0.115
10	2	0.364	0.504	0.504	3	0.200	0.556	0.567	5	0.206	0.418	0.460
13	4	0.281	0.522	0.520	2	1.000	0.512	0.512	3	0.970	0.583	0.507
15	1	0,000	0.000	0.000	1	0.000	0.000	0.000	2	0.029	0.015	0.015
21	1	0,000	0.000	0.000	2	0.130	0.506	0.506	1	0.000	0.000	0.000
23	4	0.118	0.120	0.120	3	0.318	0.448	0.470	1	0.000	0.000	0.000
25	2	0.125	0.065	0.064	4	0.077	0.641	0.643	3	0.030	0.075	0.075
26	2	0.265	0.490	0.489	2	0.000	0.290	0.290	1	0.000	0.000	0.000
36	2	0.938	0.500	0.498	3	0.905	0.585	0.551	2	0.182	0.096	0.094
56	2	0.091	0.211	0.210	2	0.182	0.486	0.486	2	0.000	0.059	0.059
Total	22	0.246 ± 0.273	0.280 ± 0.222	0.278 ± 0.222	25	0.281 ± 0.369	0.440 ± 0.185	0.440 ± 0.183	22	0.142 ± 0.301	0.136 ± 0.200	0.133 ± 0.190

Note

*A* = number of alleles; *H*_e_ = expected heterozygosity; *H*_e‐d_ = expected heterozygosity corrected by allele dosage; *H*_o_ = observed heterozygosity; *N* = number of individuals sampled.

a Voucher information and geographic coordinates for the populations are available in Appendix 1.

The transferability of 15 primer pairs was tested in four closely related taxa from the Ibero–North African group recognized within subsection *Pentasepalae* (Padilla‐García et al., 2018): *V. rosea* Desf., *V. tenuifolia* Asso subsp. *fontqueri* (Pau) M. M. Mart. Ort. & E. Rico, *V. tenuifolia* subsp. *javalambrensis* (Pau) Molero & J. Pujadas, and *V. tenuifolia* subsp. *tenuifolia*. Six individuals from different populations of each taxon were tested in agarose gel (Appendix 1). Five primer pairs were successfully amplified in all four taxa, whereas loci 13 and 21 failed in *V. rosea* individuals. Three loci exhibited no amplification in any of the tested samples, and five markers exhibited several bands or limited interspecific transferability (Table 4).

**Table 4:** Cross‐amplification tests of 15 microsatellite loci developed in *Veronica aragonensis* across four additional taxa.^a^

Locus	*V. rosea* (*N* = 6)	*V. tenuifolia* subsp. *fontqueri* (*N* = 6)	*V. tenuifolia* subsp. *javalambrensis* (*N* = 6)	*V. tenuifolia* subsp. *tenuifolia* (*N* = 6)
04	+	+	+	+
10	+	+	+	+
11	+	+	+	+
12	+	+	+	+
13	—	+	+	+
15	≡	≡	≡	≡
21	—	+	+	+
23	≡	≡	—	≡
25	—	—	—	—
26	*	*	*	*
27	+	+	+	+
29	—	—	*	*
36	≡	≡	—	—
53	—	—	—	—
56	—	—	—	—

Note

+ = successful amplification; ≡ = several bands; * = weak amplification; — = no amplification; *N* = number of individuals tested.

a Voucher information and geographic coordinates for the populations are available in Appendix 1.

## CONCLUSIONS

A new set of nuclear microsatellite loci has been developed for the tetraploid endemic species *V. aragonensis*. These markers will be useful for assessing genetic diversity and structure, as well as levels of gene flow within and among populations of this endangered endemic species. The amplification of some of these loci was successful for other closely related taxa (i.e., *V. rosea*,* V. tenuifolia* subsp. *fontqueri*,* V. tenuifolia* subsp. *javalambrensis*, and *V. tenuifolia* subsp. *tenuifolia*). Therefore, they will be suitable to provide genetic information on these additional North African and Iberian endemics.

## Appendix 1 Geographic location and voucher information for the *Veronica* samples used in this study.

**Table:**

Species	Collector no.^a^^,^ b	*N*	Locality	Collection date^c^	Latitude	Longitude	Altitude (m)	Voucher code^d^
*V. aragonensis* Stroh	NPG18^1^,^2^,^3^	34	Spain. Pyrenees. Huesca, Nerín, La Estiba mountain	25/07/2015	42°35′57.00″N	0°00′30.70″E	1728	SALA 154410
*V. aragonensis*	NPG12^2^	1	Spain. Pyrenees. Huesca, betw. Chía and Plan, Sahún mountain pass	08/07/2014	42°33′14.40″N	0°26′11.00″E	1722	SALA 154268
*V. aragonensis*	NPG13^2^	1	Spain. Pyrenees. Huesca, Bisaurri, Gabás mountain	09/07/2014	42°27′45.00″N	0°27′56.20″E	1830	SALA 154272
*V. aragonensis*	NPG15^2^	1	Spain. Pyrenees. Huesca, Seira, Barbaruens. Cotiella massif	10/07/2014	42°30′44.80″N	0°21′37.00″E	1806	SALA 154362
*V. aragonensis*	NPG22^2^	1	Spain. Pyrenees. Huesca, Yésero, Del Puerto cliff, Tendeñera mountains	14/07/2014	42°40′12.10″N	0°12′25.70″W	1971	SALA 155054
*V. aragonensis*	NPG67^2^	1	Spain. Pyrenees. Huesca, Laspuña, Ceresa mountain pass to the Peña Montañesa	27/07/2015	42°29′25.00″N	0°12′34.50″E	1713	SALA 121537
*V. aragonensis*	NPG68^2^	1	Spain. Pyrenees. Huesca, Vilas del Turbón, Turbón mountain	29/07/2015	42°24′14.30″N	0°31′34.80″E	1527	SALA 121536
*V. aragonensis*	NPG24^2^	2	Spain. Pre‐Pyrenees. Huesca, Nocito, Tozal de Guara mountain	03/08/2015	42°17′13.80″N	0°13′59.20″W	1980	SALA 121538
*V. aragonensis*	NPG25^2^	1	Spain. Pre‐Pyrenees. Huesca, betw. Arguís & Bentué de Rasal	04/08/2015	42°19′54.10″N	0°29′18.80″W	1075	SALA 121540
*V. aragonensis*	MO2047^3^	23	Spain. Pre‐Pyrenees. Huesca, betw. Arguís & Bentué de Rasal	17/07/2007	42°19′59.90″N	0°29′21.80″W	1075	SALA 121540
*V. aragonensis*	NPG28^2^,^3^	35	Spain. Granada, Puebla de Don Fadrique, La Sagra mountain	31/07/2015	37°57′12.70″N	2°33′35.60″W	2285	SALA 93529
*V. rosea* Desf.	DP783^4^	1	Morocco. Ifrane. Azrou, near Djebel Hebri	07/07/2010	33°21′10.60″N	5°08′53.40″W	1927	SALA 149323
*V. rosea*	NLG88^4^	1	Morocco. Taroudant. Souss‐Massa‐Drâa, Jebel Siroua	21/07/2013	30°46′38.40″N	7°37′5.90″W	2611	SALA 155071
*V. rosea*	VL173^4^	1	Morocco. Tinghir. Souss‐Massa‐Drâa, Ighil Mgoun	20/07/2013	31°32′11.70″N	6°16′15.00″W	3031	SALA 155074
*V. rosea*	MO5502^4^	1	Algeria. Tlemcen. Krorchef	15/06/2010	34°34′30.20″N	1°45′51.40″W	1517	SALA 149324
*V. rosea*	MO5510^4^	1	Algeria. Batna, Djebel Ichali summit	19/06/2010	35°28′18.90″N	6°10′34.50″E	1745	SALA 149338
*V. rosea*	MO5518^4^	1	Algeria. Tizi Ouzou. Djurjura Natural Park, Tizi n'Kouilal	20/06/2010	36°28′36.10″N	4°13′55.40″E	1607	SALA 149325
*V. tenuifolia* Asso subsp. *fontqueri* (Pau) M. M. Mart. Ort. & E. Rico	MO886^4^	1	Spain. Granada, betw. Calar de Sta. Bárbara & Relumbre cliff, Sierra de Baza	08/06/2000	37°22′44.50″N	2°50′30.70″W	1900	SALA 95042
*V. tenuifolia* subsp. *fontqueri*	MO1905^4^	1	Spain. Málaga, Ronda, Sierra de las Nieves	05/06/2006	36°41′41.30″N	5°00′40.60″W	1733	MGC 46659
*V. tenuifolia* subsp. *fontqueri*	MO1512^4^	1	Spain. Málaga, Ronda, Sierra de las Nieves	23/05/2002	37°41′0.00″N	5°01′0.00″W	1730	MGC 46659
*V. tenuifolia* subsp. *fontqueri*	MO1518^4^, *	1	Spain. Almería, Abla, Sierra de Baza	24/05/2002	37°22′09″N	2°50′18″W	2167	No voucher*
*V. tenuifolia* subsp. *fontqueri*	MO1519^4^	1	Spain. Almería, Dalías, Sierra de Gádor	25/05/2002	36°51′54.60″N	2°47′53.00″W	1900	SALA 120855
*V. tenuifolia* subsp. *fontqueri*	MO1520^4^	1	Spain. Almería, Dalías, Sierra de Gádor	25/05/2002	36°52′27.00″N	2°47′12.60″W	1900	SALA 120855
*V. tenuifolia* subsp. *javalambrensis* (Pau) Molero & J. Pujadas	BR222^4^	2	Spain. Salamanca, La Mata de la Armuña	20/06/2012	41°02′16.20″N	5°40′36.50″W	789	SALA 149328
*V. tenuifolia* subsp. *javalambrensis*	DP1322^4^	2	Spain. Soria, Villaciervos, El Santo	08/06/2013	41°46′08.10″N	2°38′54.60″W	1228	SALA 150477
*V. tenuifolia* subsp. *javalambrensis*	NLG05^4^	2	Spain. Guadalajara, Atienza, Ermita de Sta. Lucía	27/05/2013	41°11′23.16″N	2°52′43.02″W	1120	SALA 155105
*V. tenuifolia* subsp. *tenuifolia*	BR237^4^	1	Spain. Barcelona, Collsuspina, Sta. Coloma de Castellterçol	14/06/2013	41°49′24.00″N	2°10′36.24″E	905	SALA 155065
*V. tenuifolia* subsp. *tenuifolia*	BR241^4^	1	Spain. Huesca, Arro, S. Vitorián's monastery	17/06/2013	42°24′36.84″N	0°13′20.34″E	605	SALA 155117
*V. tenuifolia* subsp. *tenuifolia*	MO6059^4^	1	Spain. Teruel, betw. Bordón & Calanda	10/06/2013	40°41′36.60″N	0°19′9.50″W	769	SALA 155099
*V. tenuifolia* subsp. *tenuifolia*	MO6068^4^	1	Spain. Barcelona, betw. Su & Fontelles	16/06/2013	41°53′17.88″N	1°34′42.42″E	713	SALA 155121
*V. tenuifolia* subsp. *tenuifolia*	NLG09^4^	1	Spain. Barcelona, Montserrat	13/06/2013	41°36′37.00″N	1°46′13.10″E	746	SALA 155098
*V. tenuifolia* subsp. *tenuifolia*	NLG16^4^	1	Spain. Barcelona, Sta. Cecilia de Voltregà, ermita de Sta. Perpetua	15/06/2013	41°59′54.90″N	2°12′9.90″E	663	SALA 155125

Notes

*N =* number of individuals.

^a^Samples were used as follows: ^1^ = individual used for genomic library; ^2^ = individuals used for pre‐screening analyses and genotyping tests; ^3^ = individuals used for characterization of microsatellites; ^4^ = individuals used for cross‐amplification tests.

^b^BR = Blanca M. Rojas‐Andrés, collector; DP = Daniel Pinto‐Carrasco, collector; MO = M. Montserrat Martínez‐Ortega, collector; NLG = Noemí López‐González, collector; NPG = Nélida Padilla‐García, collector; VL = Víctor Lucía, collector.

^c^Date format is day/month/year.

^d^Vouchers deposited at the Universidad de Salamanca herbarium (SALA) and Universidad de Málaga herbarium (MGC).

*No voucher is available from this population due to its conservation status (Critically Endangered).
